# Spatial distribution and differences of stroke occurrence in the Rhone department of France (STROKE 69 cohort)

**DOI:** 10.1038/s41598-020-67011-8

**Published:** 2020-06-18

**Authors:** Julie Freyssenge, Florent Renard, Carlos El Khoury, Laurent Derex, Anne Termoz, Amine Chakir, Marion Douplat, Estelle Bravant, Anne-Marie Schott, Karim Tazarourte

**Affiliations:** 1 Univ. Lyon, Université Claude Bernard Lyon 1, HESPER EA 7425, F-69008 Lyon, France; 2 Emergency Department and RESCUe Network, Lucien Hussel Hospital, Lyon, France; 3 Université Jean Moulin Lyon 3 – UMR 5600 Environnement Ville Société CNRS - 18, rue Chevreul, 69007 Lyon, France; 4 Stroke Center, Department of Neurology, Hospices Civils de Lyon, F-69500 Bron, France; 5 Hospices Civils de Lyon, Public Health Department, 69500 Bron, France; 6 Hospices Civils de Lyon, Emergency Department, F-69310 Lyon, France; 7 Hospices Civils de Lyon, Emergency Department, F-69008 Lyon, France

**Keywords:** Epidemiology, Epidemiology, Epidemiology, Epidemiology, Epidemiology

## Abstract

In France, 110,000 patients are admitted to hospital per year for stroke. Even though the relationship between stroke and risk factors such as low socio-economic status is well known, research in the spatial distribution (SD) of stroke as a contributing risk factor is less documented. Understanding the geographic differences of the disease may improve stroke prevention. In this study, a statistical spatial analysis was performed using a French cohort (STROKE 69) to describe spatial inequalities in the occurrence of stroke. STROKE 69 was a cohort study of 3,442 patients, conducted in the Rhône department of France, from November 2015 to December 2016. The cohort included all consecutive patients aged 18 years or older, with a likelihood of acute stroke within 24 hours of symptoms onset. Patients were geolocated, and incidence standardized rates ratio were estimated. SD models were identified using global spatial autocorrelation analysis and cluster detection methods. 2,179 patients were selected for analysis with spatial autocorrelation methods, including 1,467 patients with stroke, and 712 with a transient ischemic attack (TIA). Within both cluster detection methods, spatial inequalities were clearly visible, particularly in the northern region of the department and western part of the metropolitan area where rates were higher. Geographic methods for SD analysis were suitable tools to explain the spatial occurrence of stroke and identified potential spatial inequalities. This study was a first step towards understanding SD of stroke. Further research to explain SD using socio-economic data, care provision, risk factors and climate data is needed in the future.

## Introduction

Stroke is a growing public health concern in developed countries^1^. In France, 110,000 patients are hospitalized per year for stroke^2^. The occurrence of the disease requires responsive management to reduce the risk of complications, making stroke prevention a priority. For this purpose, the relationship between individual risk factors, such as hypertension, diabetes, being overweight, and smoking, and stroke occurrence have been well documented^3,4^.

The risk of stroke and low socio-economic status is also well known^1,5,6^, however, research on the existence of spatial distribution (SD) and differences of stroke occurrence is still minimal. Referenced geographical data, such as the GPS coordinates and postal codes of patients or care facilities, can be used to conduct spatial and risk analysis of SD in any given living territory, and may influence towards better targeted health policies for stroke patients. Studies using statistical spatial analysis methods have been performed on other diseases^7–10^, however few have been conducted on stroke in the world^11,12^.The best-known case of significant SD analysis in stroke was the disproportionately high mortality rates measured in the 8-state region in the southeast of the United States, named the Stroke Belt^13^.

In France, to date, no studies have used this approach to characterize SD of stroke. Existing studies in the country focused on stroke hospitalization rates^14^ and mortality distribution^2,15,16^ using an epidemiological approach and a spatial statistical analysis approach respectively. SD studies have not, to date, been conducted to identify differences in stroke occurrence locations. The objective of this study was to describe the SD and geographic differences of stroke using an existing cohort and spatial health tools at a department-scale.

## Methods

### Study settings and population

The study was conducted in the Rhône department located in the Auvergne-Rhône-Alpes region in France. According to the national census, there were 1,835,903^17^ inhabitants in the department in 2016, with a population over 65 years around 16%, for an area of 3,249 km².

**Figure 1:**
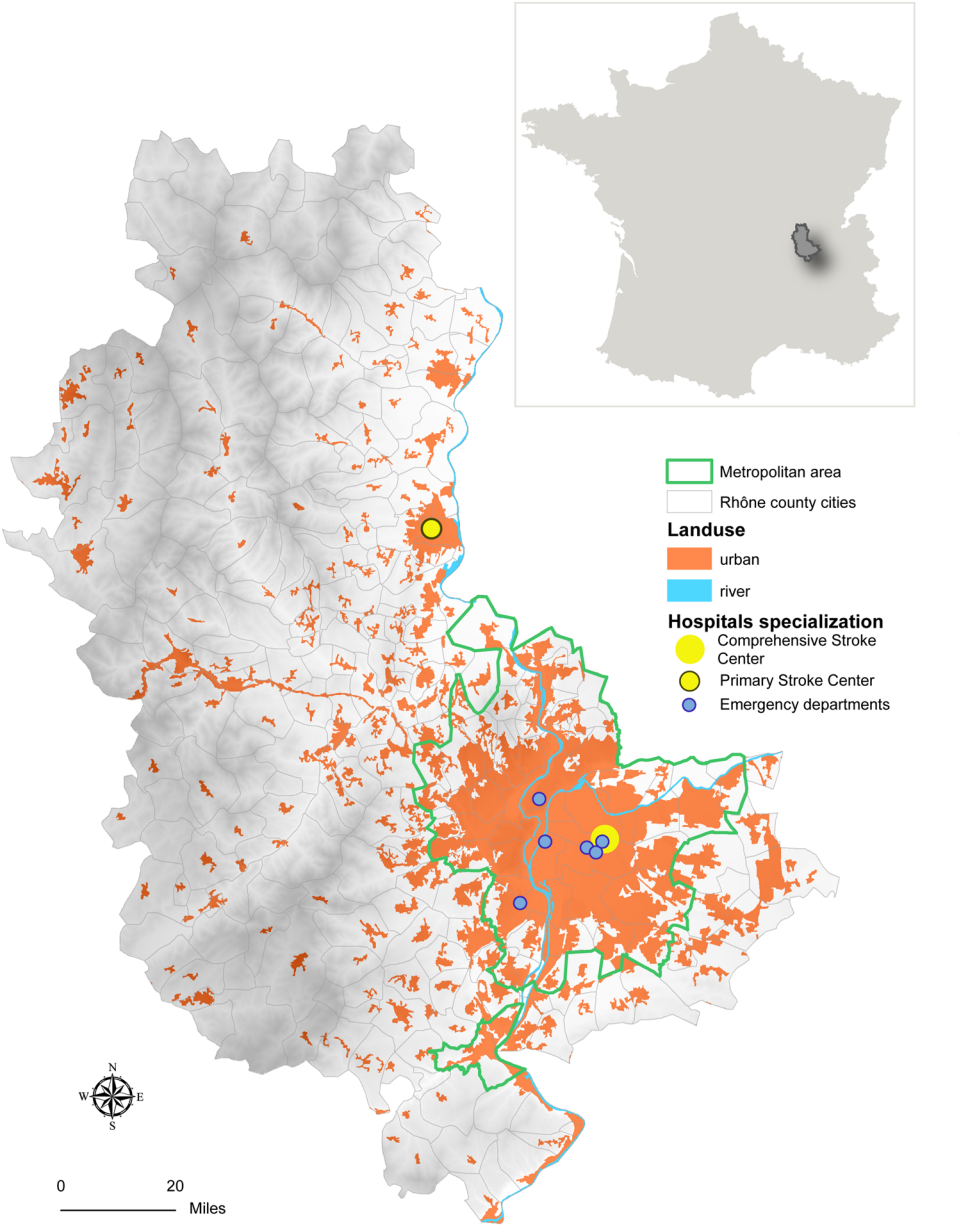
Rhône department stroke care organization and location in France (Source: CorineLandCover and STROKE 69). Maps were created using ArcGIS 10.6.1 Software (Environmental Systems Research Institute [ESRI] Inc., Redlands, CA; http://www.esri.com/).

The study was based on patients from the STROKE 69 cohort, which included all consecutive patients aged 18 years or older with the likelihood of acute stroke within 24 hours of symptoms onset, admitted to the emergency departments (EDs), a primary stroke center (PSC) or comprehensive stroke center (CSC) (Fig. 1) from November 2015 to December 2016 (n = 3,442). STROKE 69 aimed to assess the impact of previous stroke awareness campaigns on the management of acute stroke in the Rhône department.

### Ethical approval

STROKE 69 was a non-interventional study funded by the Ministry of Health. Patients were informed of the study and could refuse to participate. A non-return was considered as an informed consent. The study was approved by the ethics committee according to the French legislation and the “Commission Nationale de l’Informatique et des Libertés” (CNIL).

### Study protocol

A patient’s place of residence and the place of onset symptoms were collected in the STROKE 69 cohort. Patients were firstly located according to their place of residence, available on the STROKE 69 Case Report Form (CRF) as 82% of patients in the cohort had symptoms at home. From a patient’s home, the SAMU Centre 15, the French emergency medical service (EMS), was contacted and stroke suspected. At the request of the SAMU, patients were transferred to an ED, PSC or CSC, to have the diagnosis confirmed. The final diagnosis of a patient was then registered in the STROKE 69 CRF. All patients with a confirmed diagnosis of hemorrhagic stroke, ischemic stroke (IS) or transient ischemic attack (TIA) were included in the study. A second analysis was conducted on IS and TIA alone, since the management process for IS and TIA differs compared to hemorrhagic stroke.

The place of residence was used to analyze the incidence of stroke. Patients were geolocated with the exact address of residence and age standardization of stroke incidence rates at bundles of statistical information; Ilots Regroupés pour l’Information Statistique (IRIS) scale was performed. The IRIS are small zones grouped for statistical information and are the smallest administrative division in France with respect to demographic and geographical criteria^18^. During the time of the study, the Rhône department was divided into 769 IRIS.

### Data analysis

A protocol with predefined outcome variables was written before any analysis or inspection of the data started (https://clinicaltrials.gov/ct2/show/NCT02596607?term=NCT02596607&rank=1). The STROKE 69 dataset was geocoded based on the residential address of the stroke event using ArcGIS 10.6.1 Software (Environmental Systems Research Institute [ESRI] Inc., Redlands, CA^19^). Spatial autocorrelation analysis was conducted using ArcGIS 10.6.1. Spatial autocorrelation analysis and cluster detection were the main domain of Geographic Information System (GIS). The identification of spatial models was crucial to understand the behavior of the spatial phenomena^20^. Spatial autocorrelation methods were based on algorithms *“to determine which areas were outliers in comparison to their neighbors and could take the underlying distribution of the population into account”*^7^.

An analysis of global models was first used to describe the SD trends and to identify IRIS with a high incidence risk. Statistically significant spatial clusters were then identified at a local level. This approach characterized the SD of stroke at a territorial level, in its globality, and then locally by IRIS. At the local level, IRIS was statistically identified and represented on a map.

Two statistical tests were used at a global level. The global level was defined as the general trend given for the SD of stroke at the Rhône scale (the global territory without distinction of IRIS). This trend was expressed in the form of a spatial logic of stroke distribution: dispersed, aggregated or without spatial logic, randomly. The first test was the Moran’s Index (Moran’s I), used to measure spatial autocorrelation, which is the trend in spatial distribution. The Moran’s I indicated the level of correlation of the data, between −1 (no correlation, dispersed) and 1 (correlation, clustered). When the value was 0, the correlation was random^21^. The Moran’s I designated positive spatial autocorrelation but could not make a difference between high-intensity values aggregation (hot-spots) and low-intensity values aggregation (cold-spots). The Getis-Ord General G statistic^22^ was used as a second test to measure the clustering degree, from 0 (low values) to 1 (high values) to allow the distinction between hot and cold spots. The Getis-Ord General G assumed that the general trend of stroke in the territory was aggregated. The Getis-Ord General G represented the level of clustering of the sample at the Rhône department scale (global scale) in relation to the average values: a significant positive value corresponded to clusters of high values (many strokes) and a significant negative value corresponded to a cluster of low values (fewer strokes). According to the *p* values and *Z* scores, the results of the two tools for spatial statistics were interpreted to reject or accept the null hypothesis^23^.

At the local level, the tests were calculated by IRIS. The assumption was that IRIS were aggregated. The tests were used to determine the form of aggregation. Each IRIS was studied according to the neighboring IRIS. The IRIS that most closely resembled each other were considered aggregated. The aggregates were represented on a map, so that one could include several IRIS. First, the declination of Moran’s I, the Local Indicators of Spatial Association (LISA)^21^ was used. LISA allowed the identification of both spatial clusters of entities with features of the same magnitude and aberrant spatial points^24^. It was used to identify locations of statically significant clusters with either higher values (HH) or lower values (LL) than the average age-standardized stroke incidence rates at a statistical significance level of <0.05.

LISA also distinguished the aberrant point where a low value was mainly surrounded by high values (LH), and aberrant point which a high-intensity value was surrounded mainly by low-intensity values (HL). The second local declination was the Getis-Ord Gi*^25^, the declination of General G statistic. Getis-Ord GI* was used to identify statistically significant spatial clusters with high and low intensities, on condition that the *p* value was very small. With this method, the higher the statistically significant positive *Z* scores were, the more intense the cluster of high-intensity values (hot-spot) became. Similarly, the more intense the cluster of low-intensity values (cold-spot) were, the lower the statistically significant negative *Z* scores became.

### Ethical standards

Stroke 69 study was funded by the Ministry of Health and received ethics committee approval according to the French legislation. This study was a non-interventional study where patients were informed and free to refuse participation. It was approved by the ethical committee and the “Commission Nationale de l’Informatique et des Libertés” (CNIL).

## Results

**Figure 2:**
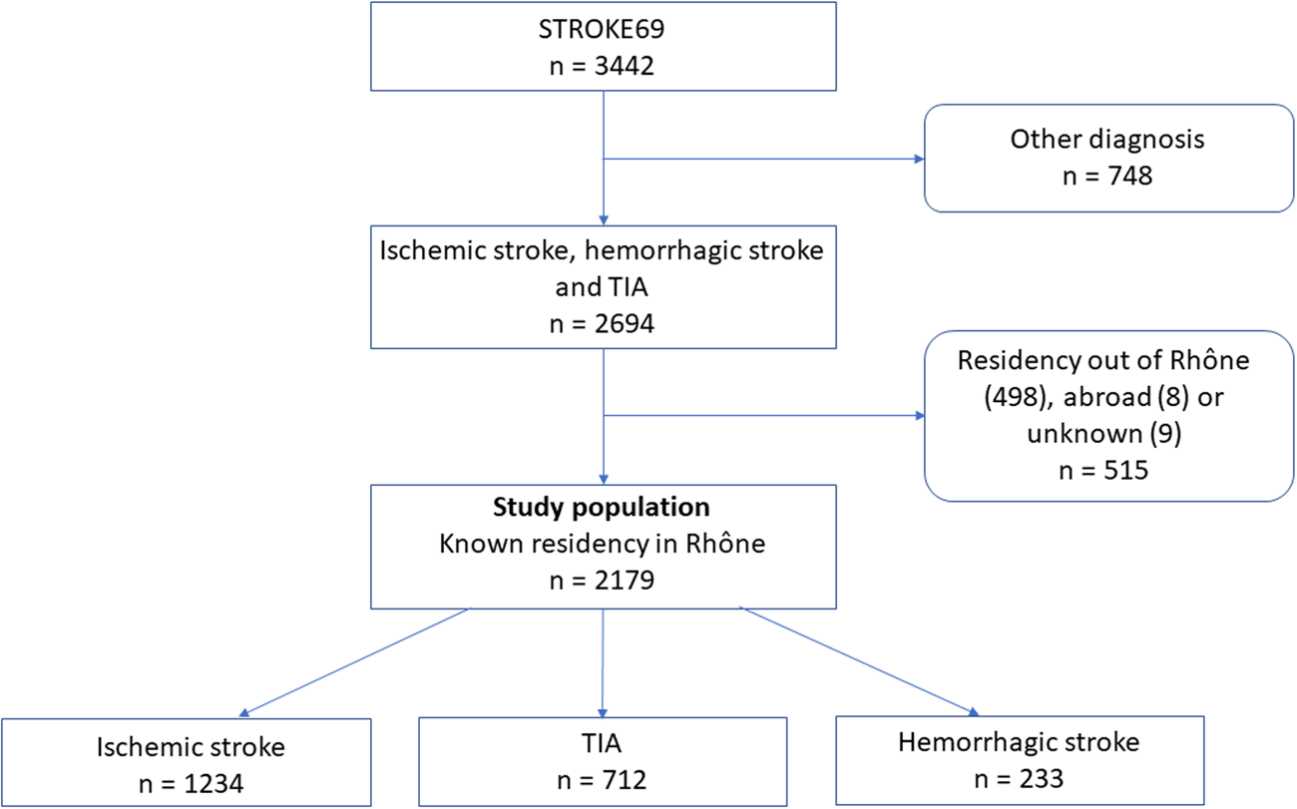
Study Flow chart of patients from the STROKE 69 cohort selected in this study according to their place of residence.

Among the 3,442 patients included in the STROKE 69 cohort, 2,694 had an IS, a hemorrhagic stroke or a TIA. Patients that were not located in the Rhône department in France, non-residents and/or patients with an unknown residential address were excluded from the study, so that the study population of 2,179 patients wasanalyzed with the spatial autocorrelation methods (Fig. 2).

There were 1,467 patients diagnosed with stroke and 712 with TIA. Strokes and TIA proportions were higher for patients over 75 years, and for both sexes. The proportion of women was higher for patients aged more than 85 years (44.8% vs 19.4% for IS; 47.3% vs 20.7% for hemorrhagic; 33.8% vs 15.6% for TIA). The mean age was lower for TIA (71.8) than stroke patients (75.5). The age-standardized stroke incidence rates per 100,000 during the period were 41.9 for IS, 8 for hemorrhagic strokes, and 24.1 for TIA (Table 1).

**Table 1:** Patients characteristics from the geographic differences and spatial distribution (SD) analysis study of stroke occurrences in Rhône county, France (STROKE 69 cohort).

	Ischemic strokes			Hemorrhagic strokes			Total strokes			TIA		
	Men	Women	Total	Men	Women	Total	Men	Women	Total	Men	Women	Total
Total, N (%)	602 (48.8)	634 (51.2)	1234	121 (51.9)	112 (48.1)	233	723 (49.2)	746 (50.8)	1467	326 (45.8)	386 (54.2)	712
Age in years, mean ± SD	71.9 ± 14.0	79.2 ± 13.8	75.7 ± 14.4	71.3 ± 14.5	77.5 ± 16.2	74.3 ± 15.7	71.8 ± 14.1	78.3 ± 14.2	75.5 ± 14.6	68.9 ± 15.8	74.3 ± 17	71.8 ± 16.7
**Age-standardized incidence rates (per 100.000)**												
All ages	42.5	41.4	41.9	8.5	7.4	8.0	51.0	48.8	49.8	22.9	25.2	24.1
<65 ans	13.4	7.6	10.5	2.9	2.0	2.4	16.3	9.6	12.9	9.6	6.9	8.3
≥ 65 ans	222.8	194.8	206.5	43.5	32.2	37.0	266.4	227.1	243.5	105.3	108.3	107.1

**Figure 3:**
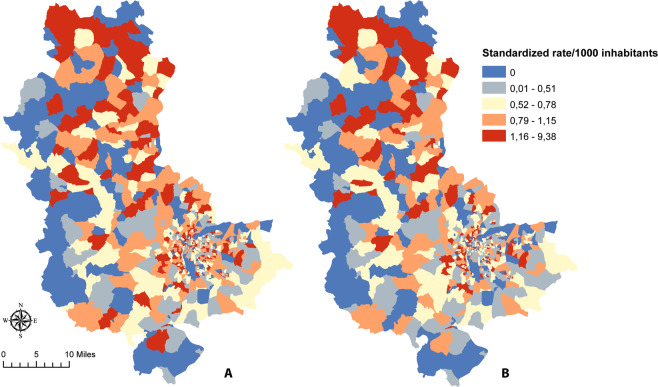
Age-standardized stroke incidence rates reported to 1,000 persons. (**A**) for all diagnosis (hemorrhagic stroke, ischemic stroke (IS) and transient ischemic attack (TIA)). (**B**) only for IS and TIA. Maps created using ArcGIS 10.6.1 Software (Environmental Systems Research Institute [ESRI] Inc., Redlands, CA; http://www.esri.com/).

In order to identify geographical patterns, the age-standardized stroke incidence rates were calculated per 1,000 people at the IRIS scale (Fig. 3). Rates were spatially lower in the south location of the department. The higher rates were located in the north. There was no significant difference between all diagnosis and only TIA/IS rates, however TIA/IS incidence had higher rates. Rates of 5.48 per 1,000 and 9.38 were measured in the cities of Ouroux and Saint-Mamert, located at the northern limit of the department.

The calculated Moran’s I for all diagnosis was 0.004, *Z* score = 1.34, and *p* value = 0.18. SD of age-standardized stroke incidence per 1,000 persons was thus random. SD was also random for only IS and TIA. Spatial relations were analyzed according to the inverse of distance (10 kms) between stroke occurrence rates. The methodology was tested with the same spatial relation conceptualization, which was the inverse of distance (10 kms) for Getis-Ord General G statistic. The sample was random with calculated Getis-Ord General G statistic (close to 0) (*Z* score = 0.16 and the *p* value = 0.87). Both methods measured the sample autocorrelation level. However, the calculation algorithms were different, so indices did not measure autocorrelation in the same way. For the second time, these results were compared at a local level.

**Figure 4:**
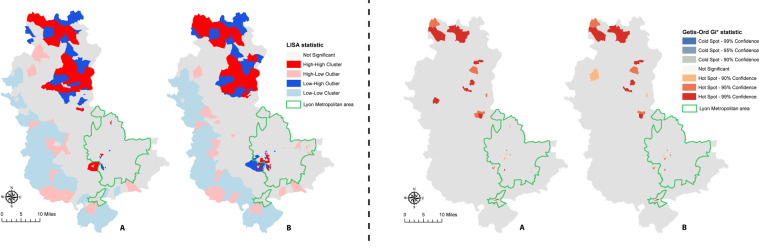
Mapping of Local Anselin Moran’s I (LISA) (left maps) and Getis-Ord Gi* (right maps) for age-standardized stroke incidence per 1,000 persons. (**A**) for all diagnosis (hemorrhagic stroke, ischemic stroke (IS) and transient ischemic attack (TIA)). (**B**) only for IS and TIA. Maps created using ArcGIS 10.6.1 Software (Environmental Systems Research Institute [ESRI] Inc., Redlands, CA; http://www.esri.com/).

The Anselin Local Moran’s I (for studying clusters and outliers) and the Getis-Ord Gi* statistic (for analyzing hot spots and cold spots) were mapped (Fig. 4) to measure autocorrelation on parts of the territory because Moran’s I and General G statistic are global spatial statistics applied to the entire area studied. Even if the distribution was random at a global level, clusters and outliers and hot and cold spots at the local level existed (Fig. 4). The maps on the left of Fig. 4 showed statistically significant clusters of high-rates incidence (HH). Maps on the right of Fig. 4 showed the hot spots. The cities in the north of the department had high rates, including a few cities in the Lyon metropolitan area and its close boundary. Based on the hypothesis that the place of residence was the place of the symptom onset, the clusters of low-rates incidence (LL) may be explained by the border effects, which were the proximity of ED and PSC located at the boundary of the Rhône department, in which patients were admitted and had faster management. These were two different methods of calculating spatial representations and SD of stroke, but cities were common to both approaches, including HH clusters and hot spots in the north of the department and the western part of the metropolitan area.

## Discussion

Analysis of the global spatial distribution of stroke in Rhône department highlighted that stroke incidence was randomly distributed. The random distribution meant there was no logical pattern to explain the incidence of stroke at a county/department level of a country. Clusters and hot spots were, however, observed when local indicators of spatial analysis methods were calculated. The general distribution logic on the department was not necessarily the same, at a local scale, as shown by LISA and Getis-Ord Gi* (Fig. 4). In addition, there was no clear difference observable between the whole sample and the only IS and TIAs sample. Thus, SD of stroke was not homogeneous and geographic differences existed.

This main strength of this study relied on its internal validity based on the statistical analysis conducted and the quality of collected data. The STROKE 69 cohort demonstrated to provide a comprehensive account of the stroke occurrence in the Rhône department, which may show the same in any given area over any given time period. The spatial model analyses were thus representative of the studied territory and represented an effective management of stroke in the Rhône department. The presence of the exact residential address of patients (identical to the occurrence place in 82% of cases) was also necessary to carry out a fine granulometric analysis of the territory at the IRIS scales. The smaller the scale, the more significant the spatial distribution analysis of the real trends in the territory were made^10,26,27^.

Another advantage of this study was the complementary use of two statistical analysis tools, which made it possible to confirm and validate the results^7,28^ for the identification and validation of clusters of high stroke rates common to both. Areas with statistically higher stroke rates were particularly relevant to identify as a first step in targeting effective preventative actions and understanding the cause of high numbers of stroke patients in those areas. This complementarity was useful for tools at a global scale: Moran’s I identified the SD trend and Getis-Ord General G statistic confirmed the results in the case of a trend towards aggregation, by specifying whether the aggregates were globally distributed in high or low values. At a local level, if clusters of high and low values (HH and LL) were identified by LISA, they would be confirmed by Getis-Ord Gi* (hot and cold spots). These techniques were robust and allowed us to identify statistically different areas.

The main study limitation was the small sample size due to the limited inclusion period for the STROKE 69 cohort study, which was not designed for spatial model analysis. This issue was particularly observed at the global level analysis based on Moran’s I and Getis-Ord General G statistics. The sample size may partly explain the non-significant results leading to a random distribution, especially since clusters at the local level still seemed to exist. The inclusion of a larger number of patients may have allowed for a greater statistical power and validation of the results from the spatial model analysis. The concern of a limited sample size could be addressed using data from a regional stroke registry, over a longer period of time, as it has been the case in France^16,26^ and in the United States to study SD of stroke mortality^11^.

Increasing the scale of analysis may however decrease the geographical accuracy in patient location and data compliance. In France, the availability of a large-scale administrative database from Hospital Discharge Diagnosis Records based on the International Classification of Disease (10^th^ revision) only included patient location at a postal code level. Additionally, the validity of medical data recorded in this database was questioned^29,30^. The risk associated with the use of these databases is based particularly on the confirmed diagnosis of stroke, at the risk of analyzing false positives and dismissing false negatives.

Another limitation to this study was the presence of clusters of low values (LL) identified by the LISA method, due to the edge effects principle. Patients were located in the IRIS situated at the border with neighboring departments. Some ED or PSC were located in these neighboring departments, but closer to patients than an ED or PSC in the Rhône department, making patient care faster. The presence of these LL clusters was therefore potentially due to the admission of these patients to an ED or PSC other than those of the Rhône department based on the hypothesis that the place of residence was the place of symptom onset and management. While not all patients with a stroke and/or residents of the Rhône department were included, the cohort still reflected the reality of management in the department. The population identified as having a high spatial logic of stroke incidence in the Rhône department, corresponded to the population that could be managed and treated in the departmental ED and PSC.

The presence of areas containing IRIS with high values raised concerns as well,. The first hypothesis based on existing literature may have linked it to the rural location^31,32^ of the clusters/hot spots, and the socio-economic level of the population^1,33^, particularly on the level of social deprivation^34,35^. Analysis, however, based on the socio-economic level have to consider the age in the choice of explanatory variables as stroke mostly affects the elderly. Similarly, and in direct link with deprivation, data on eating habits and sedentary life profiles^3,36^ of included patients could be worth collecting.

The density of the supply of care could also be considered, specifically with general practitioners to determine if there could be a relation between stroke occurrence risk and the density of general practitioners per inhabitant^37^. In addition, the spatial analysis could be supplemented by a temporal analysis of stroke incidence. A trend analysis would determine whether specific periods are more likely to result in a stroke. This would rely on climatic variables, such as air temperature^38^, air pressure or humidity^39^, or air pollutants^40^ to explain potential trends. The analysis of explanatory factors for the presence of clusters may also become the subject of future research. The identification of clusters was a necessary step in formulating hypotheses on the presence of stroke risk occurrence places, and therefore at identifying a population at risk to a worsened access to treatment.

## Conclusion

Methods of SD analysis were suitable tools at explaining stroke occurrence based on an epidemiological cohort and identifying a risk population of worsened access to treatment. The analyses conducted in this study based on the STROKE 69 cohort, at a department level in France, identified areas at risk of over incidence, particularly in the north region of the department where over incidence clusters (HH) and hot spots were observed.

To our knowledge, and to date, this was the first study based on a spatial analysis tool in France which analyzed stroke incidence. This study is a step forward towards understand SD for stroke. Further research to explain SD using socio-economic data, care provision, risk factors and climate data is needed in the future.
